# Overexpression of stathmin1 in the diffuse type of gastric cancer and its roles in proliferation and migration of gastric cancer cells

**DOI:** 10.1038/sj.bjc.6605537

**Published:** 2010-01-19

**Authors:** T-Y Jeon, M-E Han, Y-W Lee, Y-S Lee, G-H Kim, G-A Song, G-Y Hur, J-Y Kim, H-J Kim, S Yoon, S-Y Baek, B-S Kim, J-B Kim, S-O Oh

**Affiliations:** 1 Department of Surgery, Pusan National University, Beomeo-Ri, Mulgeum-Eup, Yangsan, 626–870, South Korea; 2 Department of Anatomy, Pusan National University, Beomeo-Ri, Mulgeum-Eup, Yangsan, 626–870, South Korea; 3 Medical Research Center for Ischemic Tissue Regeneration, Pusan National University, Beomeo-Ri, Mulgeum-Eup, Yangsan, 626–870, South Korea; 4 Department of Internal Medicine, Pusan National University, Beomeo-Ri, Mulgeum-Eup, Yangsan, 626–870, South Korea; 5 Department of Forensic Medicine, Pusan National University, Beomeo-Ri, Mulgeum-Eup, Yangsan, 626–870, South Korea; 6 Department of Pathology, School of Medicine, Pusan National University, Beomeo-Ri, Mulgeum-Eup, Yangsan, 626–870, South Korea

**Keywords:** stathmin1, gastric cancer, proliferation, migration

## Abstract

**Background:**

Stathmin1 is a microtubule-regulating protein that has an important role in the assembly and disassembly of the mitotic spindle. The roles of stathmin1 in carcinogenesis of various cancers, including prostate and breast cancer, have been explored. However, its expression and roles in gastric cancer have not yet been described.

**Methods:**

Stathmin1 expression in paraffin-embedded tissue sections from 226 patients was analysed by immunohistochemistry. Roles of stathmin1 were studied using a specific small interfering RNA (siRNA).

**Results:**

The expression of stathmin1 was positively correlated with lymph node metastasis, TNM stages and vascular invasion, and negatively with recurrence-free survival, in the diffuse type of gastric cancer. The median recurrence-free survival in patients with a negative and positive expression of stathmin1 was 17.0 and 7.0 months, respectively (*P*=0.009). When the expression of stathmin1 was knocked down using siRNA, the proliferation, migration and invasion of poorly differentiated gastric cancer cells *in vitro* were significantly inhibited. Moreover, s*tathmin1* siRNA transfection significantly slowed the growth of xenografts in nude mice.

**Conclusion:**

These results suggest that stathmin1 can be a good prognostic factor for recurrence-free survival rate and is a therapeutic target in diffuse-type gastric cancer.

Gastric cancer is the second most common cause of death from cancer in the world (Yuasa, 2003). The 5-year survival rate remains poor for this type of cancer (Wu *et al*, 1996, 1997; Lin *et al*, 1999). Only two survival-influencing factors, the depth of invasion and the presence of regional lymph node involvement, are commonly used in prognosis (Wu *et al*, 1996, 1997; Lin *et al*, 1999). Compared with other more extensively investigated cancers, such as breast, prostate and colon carcinomas, the molecular mechanisms involved in the transformation and progression of gastric cancer are poorly characterised. The histology of gastric carcinomas is conventionally classified into differentiated and undifferentiated types. The intestinal type is a well-differentiated tumour characterised by cohesive neoplastic cells forming gland-like tubular structures, and the diffuse type is a poorly differentiated tumour resulting in individual cells infiltrating and thickening the stomach wall (Vogiatzi *et al*, 2007). The former is frequently accompanied by liver metastasis, whereas the latter is frequently associated with peritoneal dissemination (Vogiatzi *et al*, 2007).

Stathmin is one of the microtubule-regulating proteins that have critically important roles in the assembly and disassembly of the mitotic spindle (Belmont and Mitchison, 1996; Marklund *et al*, 1996; Mistry *et al*, 1998; Mistry and Atweh, 2001, 2006). The stathmin protein family contains stathmin1 (STMN1, oncoprotein-18/Op18, metablastin), stathmin-like2 (STMN2, superior cervical ganglion-10 protein), stathmin-like3 (STMN3, superior cervical ganglion-10-like protein) and stathmin-like4 (STMN4, RaB–GAP-related protein 3) (Gavet *et al*, 1998; Singer *et al*, 2007). Stathmin1 is highly expressed in a wide variety of human cancers, including leukaemia, breast, prostate and lung cancer, and provides an attractive target for cancer therapy (Melhem *et al*, 1991; Friedrich *et al*, 1995; Brattsand, 2000; Chen *et al*, 2003). Its expression in cancer cells has been associated with their proliferation and metastasis (Mistry and Atweh, 2006; Singer *et al*, 2007). Depending on its phosphorylation state, which is mediated by p34cdc2 kinase, mitogen-activated protein kinase and other kinases, stathmin1 can regulate cell cycle through modulation of the mitotic spindle (Marklund *et al*, 1993, 1996; Luo *et al*, 1994; Larsson *et al*, 1995; Horwitz *et al*, 1997; Mistry and Atweh, 2006). Interestingly, when biopsy specimens from human prostate cancers were immunostained with an anti-stathmin1 antibody, immunoreactivity was seen in poorly differentiated tumours but not in hyperplastic prostate or highly differentiated tumours (Friedrich *et al*, 1995; Mistry and Atweh, 2006). More importantly, the level of stathmin expression correlated with the malignant behaviour of prostate cancer (Friedrich *et al*, 1995; Mistry and Atweh, 2006). Hence, it was hypothesised that the level of expression of stathmin may serve as a prognostic marker in prostate cancer.

The expression and possible roles of stathmin1 in stomach cancers have never been investigated. To reveal possible prognostic values and pathological roles of stathmin1 in gastric cancer, we examined the expression of stathmin1. We also knocked down stathmin1 expression in gastric cancer cells. We found that the expression of stathmin1 was positively correlated with lymph node metastasis, TNM stages and vascular invasion, and negatively with recurrence-free survival, in the diffuse type of gastric cancer. Moreover, knockdown of stathmin1 significantly reduced proliferation and migration of gastric cancer cells.

## Materials and methods

### Tissue specimens

Tissue samples from 226 unrelated Korean patients with primary gastric cancer, who were treated at Pusan National University Hospital, were obtained at the time of surgical resection between 1994 and 2003. Tumours and patient-matched normal epithelium were obtained at the time of surgical resection after the patients’ informed consent was obtained under a protocol reviewed and approved by the institutional review board of Pusan National University Hospital. The tissues were fixed in 10% buffered formaldehyde solution for pathological diagnosis and immunohistochemical staining. Histopathological diagnosis of each neoplastic tissue was performed according to the World Health Organization criteria by the Department of Pathology, Pusan National University Hospital. Clinicopathological staging was determined by the TNM classification. All patients had gastric cancer that was confirmed histologically, and tumour samples were checked to ensure that tumour tissue was present in more than 80% of the specimens. Follow-up data were collected until December 2008 or until the patient’s death, and the occurrence of metastasis and/or local recurrence was recorded.

### Immunohistochemistry

Immunohistochemical staining with rabbit anti-human stathmin1 polyclonal antibody (Cell Signaling Technology, Danvers, MA, USA, 1 : 50) and with rabbit anti-Ki-67 polyclonal antibody (Novocastra, Newcastle, UK, 1 : 200) was performed on 4-*μ*m sections of paraffin-embedded specimens. Briefly, after deparaffination and hydration, the slides were incubated in 0.3% H_2_O_2_ for 30 min to block endogenous peroxidase, after which the sections were blocked for 1 h at room temperature with 10% blocking serum in phosphate-buffered saline (PBS) before reacting overnight with anti-stathmin1 antibody at 4°C in a moist chamber. For Ki-67 immunostaining, antigen retrieval was performed (boiling for 20 min) in 10 mM citrate buffer (pH 6.0). After incubation with the primary antibody, the specimens were washed three times in PBS and treated with secondary antibody at room temperature for 2 h. After washing three times in PBS, the specimens were treated with ABC reagent (Dako, Carpinteria, CA, USA), followed by colour development in 3,3′-diaminobenzidine tetrahydrochloride (Dako). Finally, the slides were lightly counterstained with haematoxylin, dehydrated with ethanol, cleaned with xylene and mounted. As a negative control, duplicate sections were immunostained without exposure to primary antibodies. To quantify the stathmin1 protein expression, the mean percentage of positive tumour cells was determined by scoring at least 1000 tumour cells of at least five random fields at × 400 magnification in each section. The intensity of tumour cell staining was determined relative to that observed in adjacent endothelial cells (0, 1, 2 and 3 for negative, weak, moderate and strong). The percentage of positive tumour cells and staining intensity were then multiplied to produce a stathmin1 immunohistochemical staining score. Cases with a stathmin1 score greater than 10 were defined as positive. For Ki-67 immunohistochemistry, the number of positive tumour cells was counted in 10 representative visual fields of each xenograft. Samples were scored by two independent pathologists, neither of whom had knowledge or information pertaining to the patients’ clinical status. Statistical significance was evaluated by Fisher’s exact test, the Mann–Whitney *U*-test or Student’s *t*-test. *P*<0.05 was considered significant.

### Cell culture

The gastric cancer-derived cell lines used in this study were SNU16 and SNU638 cells. Gastric cancer-derived cell lines were grown in RPMI 1640 medium supplemented with 10% fetal bovine serum (FBS) and 50 U ml^−1^ penicillin and streptomycin (Sigma, St Louis, MO, USA). The cells were grown at 37°C in a humidified atmosphere containing 5% CO_2_. Stock cultures of each cell line were routinely sub-cultured at least once a week and the medium was changed every 2–3 days.

### siRNA and transfection

*Stathmin1* small interfering RNA (siRNA) and scrambled (SCR) siRNA were purchased from Dharmacon RNA Technologies (ON-TARGET plus, Lafayette, IN, USA) and introduced into cell lines with DharmaFECT reagents according to the manufacturer’s instructions. Dharmacon ON-TARGET plus siRNA contained four kinds of siRNA to target one gene. The sequences of *stathmin1* siRNA are as following: 5′-GAAAGACGCAAGUCCCAUG-3′ 5′-UAAAGAGAACCGAGAGGCA-3′ 5′-GAAACGAGAGCACGAGAAA-3′ and 5′-GAAGAGAAACUGACCCACA-3′.

### Cell proliferation assay

Approximately 3.0 × 10^3^ cells in 100 *μ*l of medium were plated in 96-well plates and allowed to attach for 24 h; cells were then treated with the indicated concentrations of SCR control and *stathmin1* siRNA. After 24 h, the medium was replaced with 5% FBS medium. After 5 days, 10 *μ*l per well of cell proliferation reagent WST-1 (Roche, Mannheim, Germany) was added and cells were further incubated for 2 h at 37°C, 5% CO_2_, in a humidified incubator. Absorbance of each well at 450 nm was determined using an ELISA reader (TECAN, Mannedorf, Switzerland). The cell proliferation curve was plotted using the absorbance at each time point. Viability was determined in quintuplicate.

### Cell migration assay

A modified Boyden chamber (Neuro Probe, Gaithersburg, MD, USA) was used. The pore size of polycarbonate filters was 8.0 *μ*m. The bottom chamber of the transwell chamber was filled with 30 *μ*l RPMI containing 10% FBS. At 2 days after transfection with SCR or *stathmin1*-siRNA, cells were trypsinised. The cells were then suspended at a density of 1 × 10^6^ cells per ml in 50 *μ*l of RPMI supplemented with 0.5% FBS and placed in the upper chamber. The cells were incubated 2 h at 37°C in 5% CO_2_. After the upper side of the filter was scraped with a cotton tip to eliminate cells that had not migrated through it, the filter was removed and fixed in methanol before staining with Diff Quik Solution (Sysmex, Kobe, Japan). The cell number in 10 randomly chosen fields was determined using a light microscope. Experiments were performed in triplicate and repeated three times.

### Matrigel invasion assay

At 1 day after transfection with SCR or stathmin1 siRNA, cells were trypsinised. The cells were then suspended at a density of 5 × 10^4^ cells per ml in 500 *μ*l of RPMI supplemented with 0.5% FBS and mitomycin C (0.01 *μ*g ml^−1^, Sigma), added to 8-*μ*m porous BioCoat Matrigel chamber inserts (BD Biosciences, San Jose, CA, USA) and placed in wells filled with 0.7 ml of medium supplemented with 10% fetal calf serum as chemoattractant. After 2 days of incubation, the upper side of the filter was scraped with a cotton tip to eliminate cells that had not migrated through it. The invasive ability of the cells was determined by counting the cells that had migrated to the lower side of the filter with a microscope. Experiments were performed in triplicate, and at least 10 fields were counted in each experiment.

### Western blotting

Total protein extracts were separated by 10–12% SDS–PAGE (20–50 *μ*g per lane), and electro-transferred to polyvinylidene fluoride membranes. Anti-stathmin1 (1 : 500, Cell Signaling Technology) and anti-*β*-actin (1 : 1000, Abcam, Cambridge, UK) antibodies were diluted in PBS/T (PBS/tween; 5% milk powder) and incubated with the membranes at 4°C overnight. The appropriate secondary antibody was applied (1 : 2000, horseradish peroxidase-conjugated anti-mouse and anti-rabbit) at room temperature for 1 h. Immunoreactive proteins were visualised by enhanced chemiluminescence (Amersham Bioscience, Freiburg, Germany).

### RNA preparation, complementary DNA synthesis and quantitative real-time PCR

Total RNA was isolated from SNU638 cell lysates using an RNeasy Mini Kit (Qiagen, Hilden, Germany) according to the manufacturer’s instructions. Total RNA was then treated with DNase I in the presence of anti-RNase (Ambion, Austin, TX, USA) to remove DNA contamination before complementary DNA synthesis. The complementary DNA was synthesised with random primers (Roche, Basel, Switzerland) and avian myeloblastosis virus reverse transcriptase (Promega, Madison, WI, USA). Real-time PCR (power SYBR Green, ABI, Warrington, UK) analysis was performed using an ABI Prism 7500 Sequence Detector according to the manufacturer’s protocol. Primer sequences were as follows: for stathmin1, 5′-CCCCTTTCCCCTCCAAAGAA-3′ (forward), 5′-TCGCAAACGTTCCAGTTTGG-3′ (reverse); and for *β*-actin, 5′-ATCATGTTTGAGACCTTCAA-3′ (forward), 5′-CATCTCTTGCTCGAAGTCCA-3′(reverse). Fold changes for the genes of interest were calculated after normalisation with the endogenous control *β*-actin and using the comparative threshold cycle (*C*_t_) method. These experiments were performed in triplicate and repeated in three independent experiments.

### Xenograft assay

The SNU638 cells were transfected with SCR or *stathmin1* siRNA. After 2 days, cells were collected by trypsinisation and washed twice before injection. Cell vitality was >95% as determined by trypan blue dye exclusion. Cells (2 × 10^6^ cells in 100 *μ*l PBS) were injected subcutaneously into nude mice. All injected mice formed tumours. Tumour volumes were measured every week from week 3 to week 7 and calculated using the following formula: 0.5236 × L1 × (L2)^2^, where L1 is the long axis and L2 is the short axis of the tumour (Qian *et al*, 2007). After 7 weeks, mice were killed. Tissues were fixed in 10% buffered formaldehyde solution and paraffin blocks were obtained. Procedures related to animal care were in accordance with the guidelines of the ‘Guideline for the Welfare of Animals in Experimental Neoplasia’(Workman *et al*, 1998). The Pusan National University Institutional Animal Care and Use Committee (PNUIACUC) approved the experimental procedures.

### Terminal deoxynucleotidyl transferase dUTP nick end-labelling assay

Apoptosis was evaluated using the terminal deoxynucleotidyl transferase dUTP nick end-labelling assay. The terminal deoxynucleotidyl transferase dUTP nick end-labelling assay was performed using the *In Situ* Apoptosis Detection Kit (Chemicon, Temecula, CA, USA) according to the manufacturer’s instructions. Apoptotic cells were visualised under fluorescent microscope (Olympus BX50, Tokyo, Japan). The number of apoptotic cells as a percentage of the total number of cells was calculated on the basis of data obtained from 10 random areas. Each experiment was repeated four times.

### Statistical analysis

Statistical comparison between two groups was performed using the non-parametric Mann–Whitney *U*-test or Student’s *t*-test. For comparison of more than three groups, we used one-way analysis of variance, followed by Tukey’s multiple comparison. *P*-values <0.05 were considered statistically significant. The overall survival time was defined as the interval between the date of treatment and the date of death or until the last objective follow-up information was obtained. Recurrence-free survival time was regarded as the time interval between tumour treatment and detection of the first locoregional recurrence and/or distant metastasis or the date of last follow-up, whichever occurred first. Recurrence-free survival according to stathmin1 overexpression was constructed using the Kaplan–Meier method. Curves were compared by the log-rank test at 95% significance. Multivariate analysis was carried out using the Cox regression method. A *P*-value <0.05 was considered to be statistically significant. Data were analysed using SPSS software, version 12.0 (SPSS, Chicago, IL, USA).

## Results

### Immunohistochemical analysis of stathmin1^+^ cells in gastric cancer

**Figure 1:**
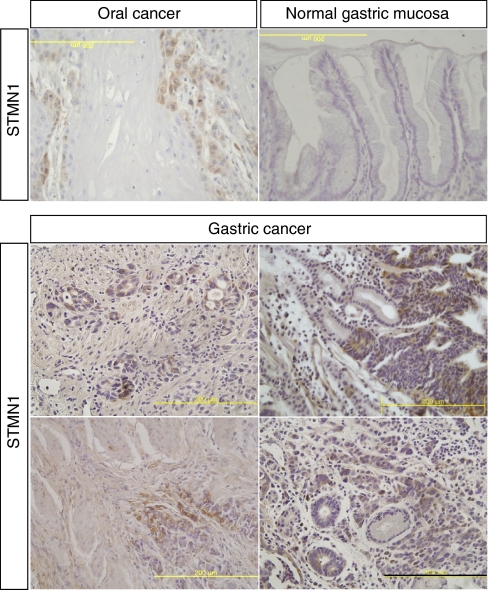
Immunohistochemical staining of stathmin1 in oral and gastric cancer sections. Anti-stathmin1 antibody was used for immunohistochemical staining in human oral and gastric cancer tissues as described in ‘Materials and Methods’ section. The invading oral cancer cells were positive for stathmin1 protein in the cytoplasm. The normal gastric mucosal cells were either negative or very weakly positive for stathmin1 protein in the cytoplasm. The invading gastric cancer cells were positive for stathmin1 protein. Scale bar, 200 *μ*m.

**Figure 2:**
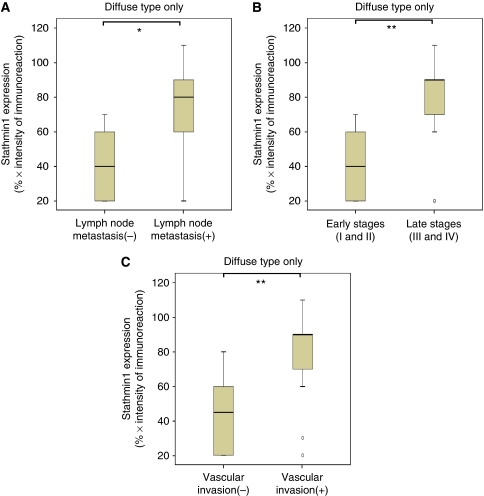
Correlationship between the stathmin1 expression level and clinicopathological characteristics. Data of stathmin-positive patients with diffuse-type gastric cancer are presented. (**A**) The state of stathmin1 protein expression in patients with lymph node metastasis in diffuse type gastric cancer (*n*=17) and in patients without metastasis (*n*=7). Stathmin1 protein expression in patients with lymph node metastasis is significantly higher than in patients without lymph node metastasis (^*^P<0.05, Mann–Whitney *U*-test). (**B**) The state of stathmin1 protein expression in patients with early stages (I and II) of diffuse type gastric cancer (*n*=9) and patients with advanced stages (III and IV) of diffuse-type gastric cancer (*n*=15). Stathmin1 protein expression in advanced stages is significantly higher than that in early stages (^**^P<0.01, Mann–Whitney *U*-test). (**C**) The state of stathmin1 protein expression in patients with vascular invasion in diffuse-type gastric cancer (*n*=14) and in patients without vascular invasion (*n*=10). Stathmin1 expression in patients with vascular invasion is significantly higher than in patients without vascular invasion (^**^P<0.01, Mann–Whitney *U*-test).

To characterise stathmin1 expression in human gastric cancer, we used archival paraffin-embedded tissue sections (total 226 patients) for immunohistochemistry. A rabbit anti-stathmin1 antibody (Cell Signaling Technology) was evaluated and then recommended for both western blot and immunohistochemistry by the manufacturer. To evaluate the anti-stathmin1 antibody in our lab, we performed immunohistochemistry using oral squamous cancer tissue. Stathmin1 was expressed in invading oral cancer cells (Figure 1A), which was consistent with the previous report (Kouzu *et al*, 2006). In normal gastric mucosa, it was difficult to detect stathmin1 expression; however, we clearly observed cytoplasmic expression of stathmin1 in gastric cancer tissue and in metastatic gastric cancer cells (Figure 1). Stathmin1 was expressed in 25.5 and 76.5% of diffuse and intestinal types of gastric cancer tissues, respectively. The correlation between the clinicopathological characteristics of patients with gastric cancer and the status of stathmin1 expression is summarised in Table 1. Interestingly, in the diffuse type of gastric cancer, stathmin1 expression was correlated with N staging (*P*=0.008; Table 1). The stathmin1 expression level was also significantly higher in the gastric cancer group with lymph node metastasis than in the group without lymph node metastasis (Mann–Whitney *U*-test, *P*=0.013, Figure 2). Moreover, stathmin1 expression correlated with the TNM stage grading of the diffuse type of gastric cancer (*P*=0.005; Table 1). The stathmin1 expression level was also significantly higher in the group with advanced gastric cancer than in the group with early-stage cancer (Mann–Whitney *U*-test, *P*=0.002, Figure 2). Furthermore, stathmin1 expression correlated with vascular invasion in diffuse-type gastric cancer (*P*=0.006; Table 1). The stathmin1 expression level was also significantly higher in the group with vascular invasion than in the group without vascular invasion (Mann–Whitney *U*-test, *P*=0.003, Figure 2).

### Prognostic significance of stathmin1 expression in gastric cancer

**Figure 3:**
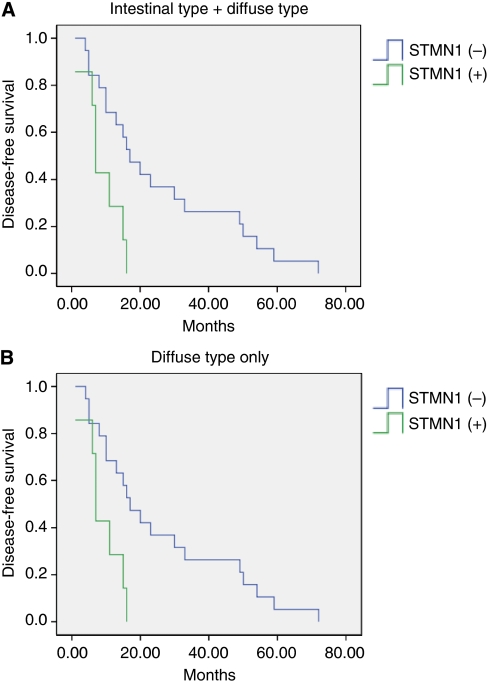
Kaplan–Meier curves for recurrence-free survival according to stathmin1 overexpression in patients with gastric cancer ((**A**) diffuse type+intestinal type, *P*<0.05; (**B**) diffuse type only, *P*<0.01). When stathmin1 overexpression was observed, the recurrence-free survival rate decreased significantly, especially in patients with diffuse-type gastric cancer.

To evaluate stathmin1 expression as a prognostic factor, we performed survival analysis using the Kaplan–Meier method. The overall survival curves in relation to stathmin1 expression were not significant (data not shown). However, the recurrence-free survival curves in relation to stathmin1 expression were significant (*P*=0.049, Figure 3). The mean recurrence-free survival was 25.1 months (median 17.0 months) in the stathmin1-negative group and 17.4 months (median 11.0 months) in the stathmin1-positive group. Interestingly, in the diffuse type of gastric cancer, the relationship between stathmin1 expression and recurrence-free survival was more significant (*P*=0.009, Figure 3). The mean recurrence-free survival was 25.9 months (median 17.0 months) in the stathmin1-negative group and 9.0 months (median 7.0 months) in the stathmin1-positive group. When we analysed the recurrence-free survival of the earlier case groups (stages 1–2) to avoid the influence of stage deviation, the difference was still significant (*P*=0.042). Prognostic significance of stathmin1 expression in the diffuse type of gastric cancer was further confirmed by multivariate survival analysis (Table 2).

### Roles of stathmin1 in gastric cancer cells

**Figure 4:**
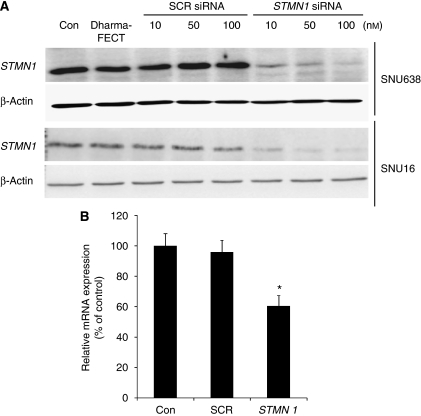
Stathmin1 was specifically downregulated by *stathmin1*siRNA. Cells were transfected with scrambled siRNA or *stathmin1* siRNA. (**A**) Two days later, SNU638 and SNU16 cells were collected and stathmin1 protein levels were detected by western blotting. *β*-actin expression was monitored for normalisation. (**B**) SNU638 cells were collected and *stathmin1* mRNA levels were examined by real-time PCR. Values are expressed as the percentage of control, which was defined as 100% (*n*=6). Results were analysed by one-way ANOVA, followed by Tukey's multiple comparison test. ^*^P<0.01 *vs* control. mRNA, messenger RNA; SCR, scrambled; siRNA, small interfering RNA.

**Figure 5:**
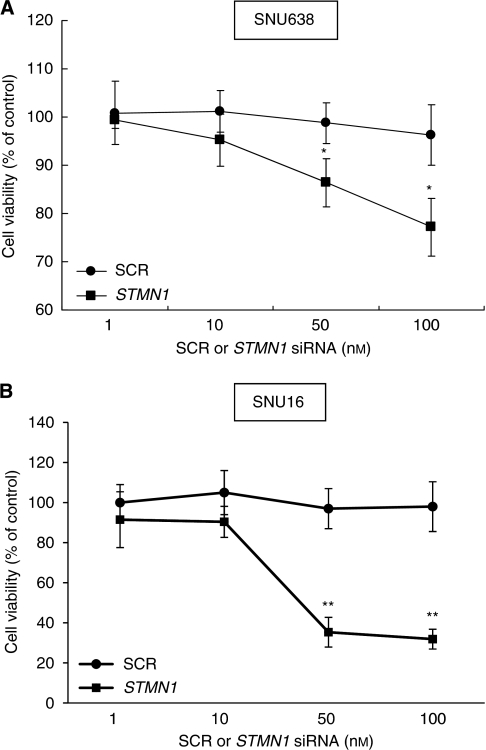
Effect of stathmin1 silencing on proliferation of gastric cancer cells. SNU638 (**A**) or SNU16 (**B**) cells were transfected with SCR siRNA or *stathmin1* (*STMN1*) siRNA. After 5 days of incubation, cell proliferation was evaluated in WST assay. Data are expressed as percentage change (means±s.d.) compared with controls and represent four independent experiments. (^*^P<0.05, ^**^P<0.01 *vs* SCR siRNA, one-way ANOVA followed by Tukey's multiple comparison). ANOVA, one-way analysis of variance; SCR, scrambled; siRNA, small interfering RNA.

**Figure 6:**
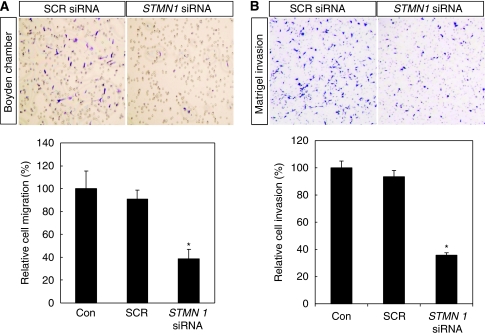
Effect of stathmin1 silencing on the migration and invasion of gastric cancer cells. (**A**) Cell migration was evaluated in the Boyden migration assay two days after SNU638 cells were transfected with scrambled (SCR) small interfering RNA (siRNA) or *stathmin1* siRNA. (**B**) Cell invasion was evaluated in the Matrigel invasion assay as described in the ‘Materials and Methods’ section. Data are expressed as percentage change (means±s.d.) compared with controls and represent four independent experiments. (^*^P<0.01 *vs* SCR siRNA, one-way analysis of variance (ANOVA) followed by Tukey's multiple comparison). Representative microscopic images were presented in the upper panel of each assay graph. ANOVA, one-way analysis of variance; SCR, scrambled; siRNA, small interfering RNA.

To examine the possible roles of stathmin1 in gastric cancer cells, we knocked down the expression of stathmin1 using siRNA. Before siRNA experiments, we confirmed stathmin1 expression in gastric cancer cell lines by western blotting (data not shown). To confirm the efficacy of *stathmin1* siRNA, we performed real-time PCR and western blotting (Figure 4). When SNU638 and SNU16 cells were transfected with *stathmin1* siRNA, the expression of stathmin1 was almost completely abolished at the protein level (Figure 4A). Moreover, the messenger RNA level was also significantly reduced by siRNA (Figure 4B). To examine the role of stathmin1 in proliferation, we conducted WST assays after the transfection of *stathmin1* siRNA. *Stathmin1* siRNA significantly reduced the proliferation of SNU638 and SNU16 cells compared with SCR siRNA at 100 nM (Figure 5A and B). We next examined the role of stathmin1 in cancer cell migration. We observed a significant difference between cells transfected with SCR or *stathmin1* siRNA in the migration assay (Figure 6A). Moreover, cancer cell invasion was also significantly reduced by *stathmin1* siRNA in the Matrigel invasion assay (Figure 6B).

**Figure 7:**
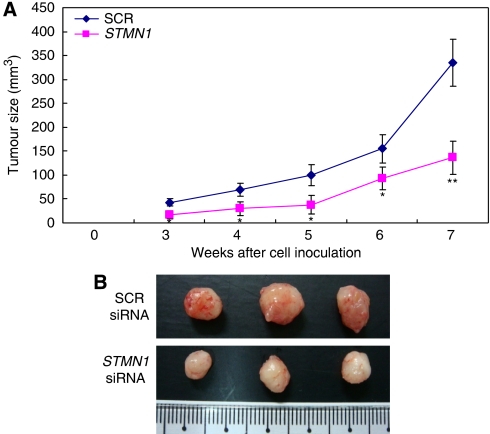
Effect of stathmin1 silencing on *in vivo* tumour growth. (**A**) SNU638 cells were transfected with scrambled (SCR) small interfering RNA (siRNA) or *stathmin1* (*STMN1*) siRNA, and nude mice were inoculated subcutaneously with 2 × 10^6^ cells at two sites per mouse. The tumour mass (xenograft) volume was measured every week from week 3 to week 7. Data are expressed as the means±s.d. and represent four independent experiments. (^*^P<0.05, ^**^P<0.01 *vs* SCR siRNA, one-way analysis of variance (ANOVA) followed by Tukey's multiple comparison) (**B**) The tumour masses (xenograft) were dissected out 7 weeks later and presented. ANOVA, one-way analysis of variance; SCR, scrambled; siRNA, small interfering RNA.

To confirm this effect of *stathmin1* siRNA *in vivo*, we subcutaneously inoculated nude mice with SNU638 cells transfected with SCR or *stathmin1* siRNA. We observed significantly slower growth of cancer cells transfected with *stathmin1* siRNA than of cancer cells transfected with SCR siRNA (Figure 7). When we examined the tumour tissues of xenografts, the expression of stathmin1 was significantly reduced in the *stathmin1* siRNA group compared with the SCR group (Supplementary Figure 1). Moreover, proliferation was also reduced in the *stathmin1* siRNA group (Supplementary Figure 2). In contrast, apoptosis was significantly increased in the *stathmin1* siRNA group compared with the SCR group (Supplementary Figure 3).

## Discussion

Stathmin1 is expressed in the more poorly differentiated types of several cancer tissues. In prostate cancer, stathmin1 expression is similar in Gleason patterns 3 and 4; however, a significant increase in stathmin1 levels occurs in Gleason pattern 5. Similarly, greater expression of stathmin1 was observed in androgen-independent cancer cells than in androgen-dependent cancer cells (Friedrich *et al*, 1995). In breast cancer, stathmin1 levels negatively correlated with estrogen receptor expression and positively correlated with a high fraction of aneuploid cells, proliferation, tumour size and histopathological grade (Brattsand, 2000). In this study, we characterised stathmin1 expression in gastric cancer. In normal mucosa, stathmin1 was rarely detected. Interestingly, significant association of stathmin1 expression with more advanced stages, lymph node metastasis and vascular invasion was observed in the diffuse type of gastric cancer. Stathmin1 was also detected in invading gastric cancer cells. Moreover, the association of stathmin1 expression with the recurrence-free survival rate was more significant in the diffuse type of gastric cancer. Furthermore, in this study, functional significance of stathmin1 was demonstrated in SNU638 and SNU16 cells. These results suggest that stathmin1 might be a good prognostic factor in the diffuse type of gastric cancer.

Critical roles for stathmin1 in the proliferation of cancer cells, such as prostate cancer and osteosarcoma cells, have been demonstrated (Mistry and Atweh, 2006; Wang *et al*, 2007). In this study, we show, to the best of our knowledge, for the first time that stathmin1 regulates proliferation of poorly differentiated gastric cancer cells. Two mechanisms for stathmin1 regulation of microtubules and proliferation have been suggested (Rubin and Atweh, 2004). Stathmin1 can bind two unpolymerised tubulin heterodimers and form a ternary stathmin–tubulin complex. This tubulin-sequestrating activity of stathmin prevents microtubule growth by diminishing the intracellular pool of tubulin that is available for polymerisation. The other mechanism of stathmin regulation of proliferation is a catastrophe-promoting activity. Stathmin binds tubulin heterodimers at the microtubule ends and increases the rate of catastrophe through a GTP hydrolysis-dependent mechanism.

The importance of stathmin1 in cell migration has been demonstrated (Ozon *et al*, 2002; Jin *et al*, 2004; Giampietro *et al*, 2005). Stathmin1 downregulation *in vivo* in both mice and *Drosophila* decreased the motility of proliferating neurons and germ and border cells. Moreover, stathmin1 is overexpressed in metastatic *vs* clinically localised prostate carcinomas, invasive recurrent hepatocarcinomas, advanced mammary carcinomas and recurrent/metastatic sarcomas. These results suggest that increased stathmin1 expression and activity stimulates cancer cell migration *in vitro*, *in vivo* and in human cancers. In this study, we examined the possible roles of stathmin1 in the migration of gastric cancer cells because it was expressed in invading gastric cancer cells. In agreement with previous reports, we observed a significant difference in the migration of SCR and *stathmin1* siRNA-transfected cells. Regulation of cancer cell migration by stathmin1 may explain the greater lymph node metastasis and poor prognosis in gastric cancer patients with stathmin1 overexpression.

The effects of stathmin1 silencing on proliferation and migration of gastric cancer cells in this study were not complete, although protein expression of stathmin1 was almost completely abolished. This may be due to other microtubule integrity modifiers, such as other stathmin family members (STMN2–4), which may compensate for the lack of stathmin1 expression after siRNA-mediated silencing.

In this study, we showed for the first time that stathmin can be used as a good prognostic factor and therapeutic target in the diffuse type of gastric cancer. In future studies, it needs to be examined whether stathmin1 silencing can increase the responsiveness of gastric cancer cells to different anti-cancer drugs.

## Figures and Tables

**Table 1:** Correlation between the expression of stathmin1 and clinical classification in gastric cancer

	**Immunoreactivity case No. (%)**			
**Clinical classification**	**Total**	**Negative**	**Positive**	*P*-value*
*Age at surgery (years)*				0.202
<60	120	60 (50.0)	60 (50.0)	
60⩽, <70	68	25 (36.7)	43 (63.3)	
70⩽	38	16 (42.1)	22 (57.9)	
				
*Gender (diffuse type)*				0.628
Male	43	31 (72.1)	12 (27.9)	
Female	51	39 (76.5)	12 (23.5)	
				
*Gender (intestinal type)*				0.464
Male	105	24 (22.9)	81 (77.1)	
Female	27	8 (29.6)	19 (70.4)	
				
*N stages (diffuse type)*				0.008
N0∼N1	70	57 (81.4)	13 (18.6)	
N2–N3	24	13 (54.2)	11 (45.8)	
				
*N stages (intestinal type)*				1.000
N0∼N1	116	27 (23.3)	89 (76.7)	
N2–N3	16	4 (25.0)	12 (75.0)	
				
*TNM stage (diffuse type)*				0.005
1∼2	58	49 (84.5)	9 (15.5)	
3∼4	36	21 (58.3)	15 (41.7)	
				
*TNM stage (intestinal type)*				0.946
1∼2	107	25 (23.4)	82 (76.6)	
3∼4	25	6 (24.0)	19 (76.0)	
				
*Vascular invasion (diffuse type)*				0.006
Negative	61	51 (83.6)	10 (16.4)	
Positive	33	19 (57.6)	14 (42.4)	
				
*Vascular invasion (intestinal type)*				0.634
Negative	98	22 (22.4)	76 (77.6)	
Positive	34	9 (26.5)	25 (73.5)	

Abbreviation: TNM=The tumour-node-metastasis staging system. ^*^P<0.05 was defined as significant, Fisher's exact test.

**Table 2:** Univariate and multivariate analysis of prognostic factors in the diffuse type of gastric cancer patients for recurrence-free survival

	**Univariate analysis**	**Multivariate analysis**	
	***P*-value**	***P-*value**	**RR (95% CI)**
Age (years)	0.508	0.694	0.837 (0.345-2.033)
<60 (*vs* ⩾60)			
Gender	0.090	0.850	1.104 (0.395-3.088)
Male (*vs* female)			
Lymph node metastatsis	0.010	0.230	0.488 (0.151-1.576)
N2+N3 (*vs* N0+N1)			
TNM stage	0.024	0.349	0.576 (0.181-1.829)
StageIII+IV (*vs* stageII+I)			
Stathmin1 expression	0.009	0.049	0.328 (0.108-0.996)
Positive (*vs* negative)			

Abbreviations: CI=confidence interval; RR=relative risk; TNM=The tumour-node-metastasis staging system.
