# Supplementary figures and images for: Overexpression of stathmin1 in the diffuse type of gastric cancer and its roles in proliferation and migration of gastric cancer cells
Source: Br J Cancer. 2010 Jan 19;102(4):710–8. doi: 10.1038/sj.bjc.6605537 (PMC2837578; doi:10.1038/sj.bjc.6605537)

Supplementary Figure 1

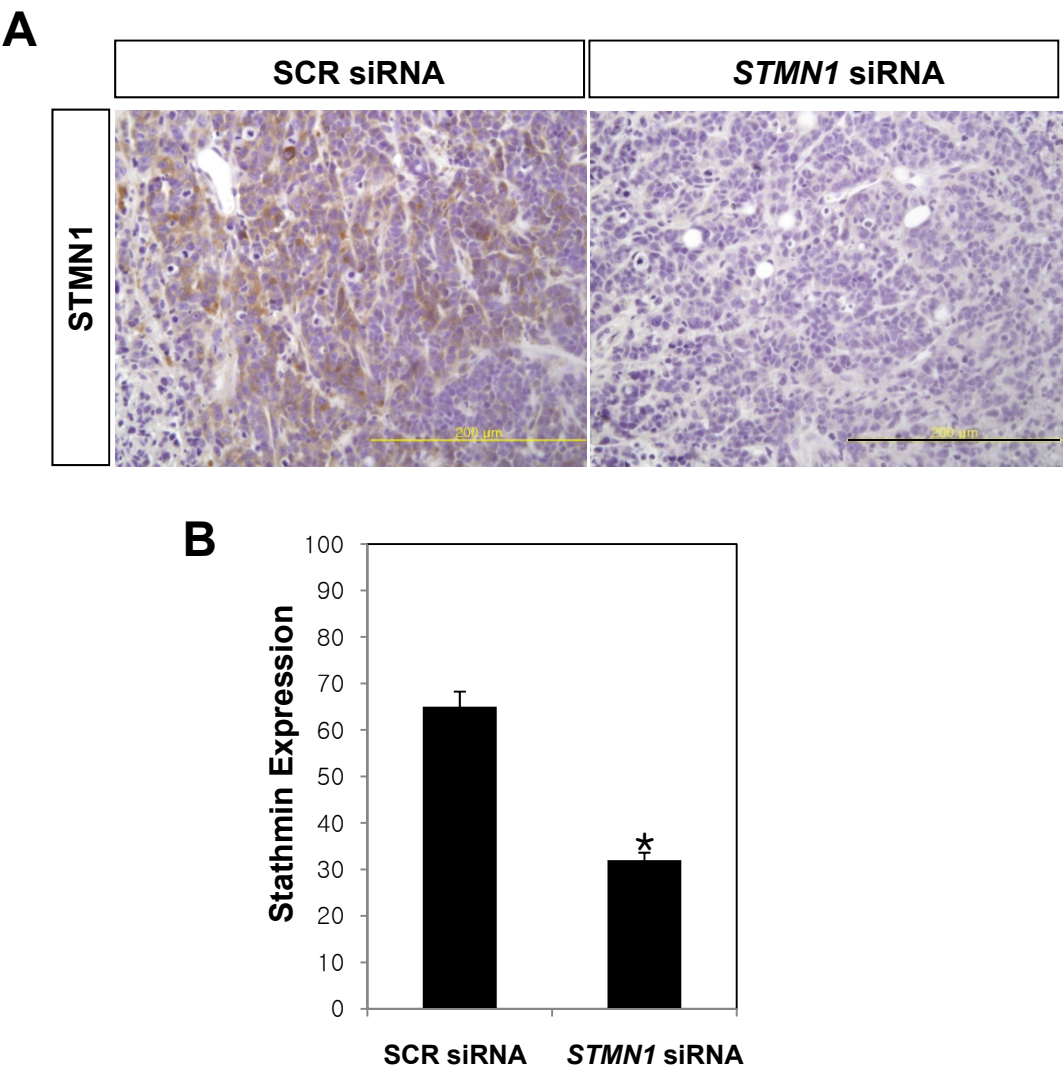

Supplementary Figure 2

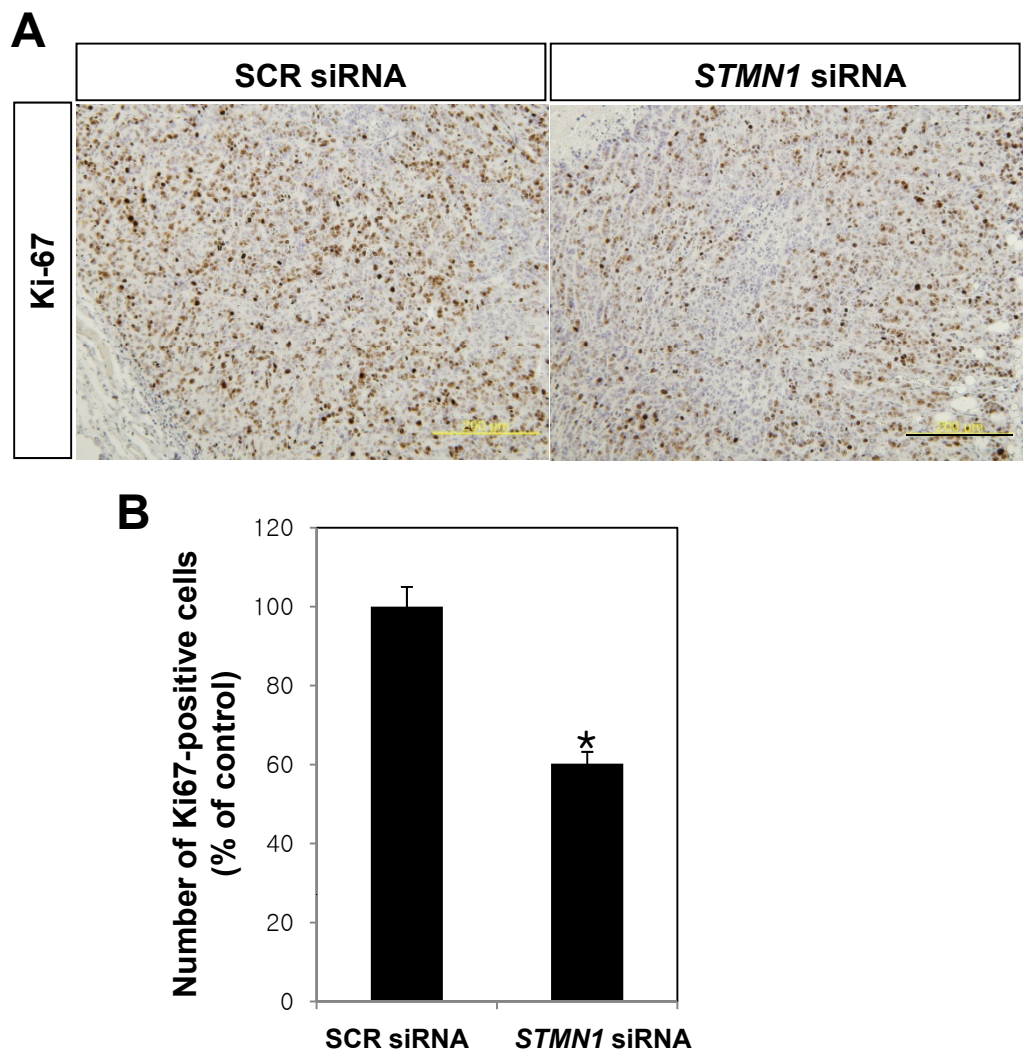

Supplementary Figure 3

**A**

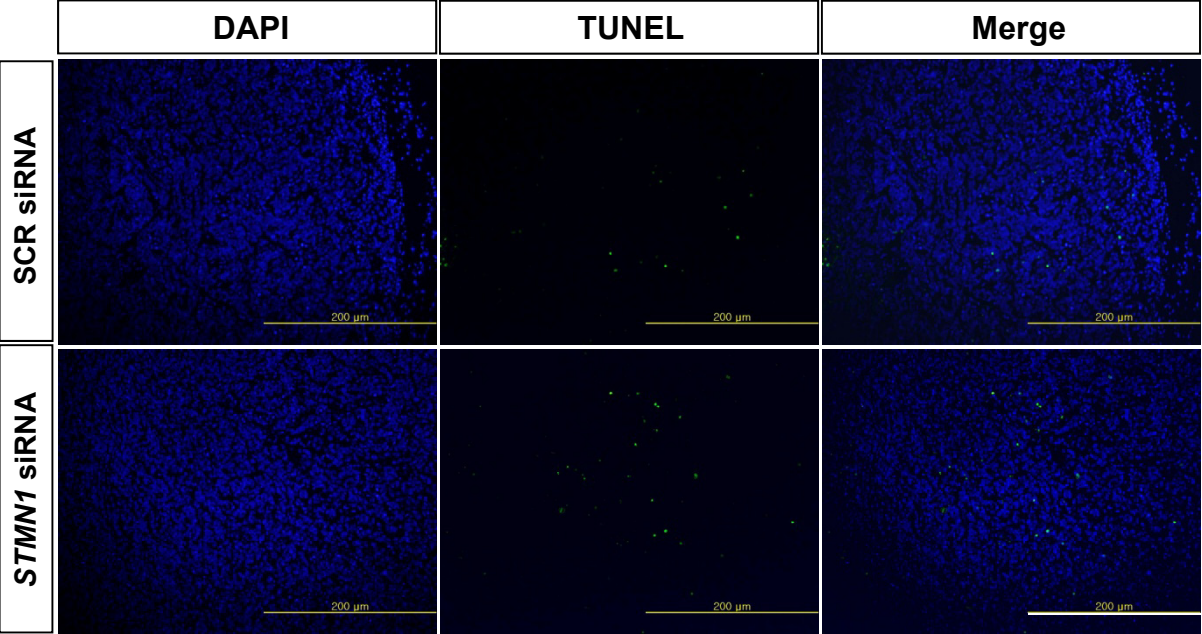

**B**

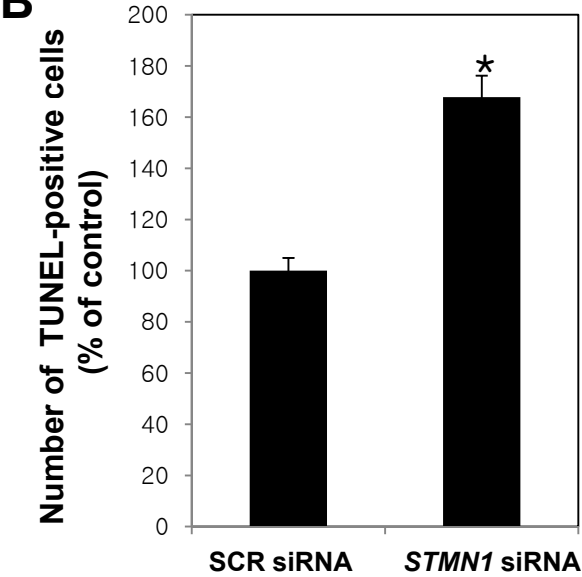

Supplement: Supplementary Figures [file 6605537x1.pdf]
