# Supplementary material for: Overexpression of stathmin1 in the diffuse type of gastric cancer and its roles in proliferation and migration of gastric cancer cells
Source: Br J Cancer. 2010 Jan 19;102(4):710–8. doi: 10.1038/sj.bjc.6605537 (PMC2837578; doi:10.1038/sj.bjc.6605537)
Supplement: Supplementary Figure Legends [file 6605537x2.doc]

**Supplementary Figure 1**. State of stathmin1 protein expression in xenografts. Gastric cancer cell transplantation into nude mice was performed as described in the legend of Figure 7. The tumour masses (xenograft) were dissected and fixed in 10% buffered formaldehyde solution. Stathmin1 immunohistochemistry was performed as described in “Materials and Methods” section. Data are expressed as means±SD and represent four independent experiments (**P*<0.01 vs. SCR siRNA, Student *T*-test). Scale bar, 200 µm.

**Supplementary Figure 2**. Proliferation of tumour cells was reduced by *stathmin1* siRNA in xenografts. Xenograft tissues described in the legend of Supplementary Figure 1 was used for Ki-67 immunostaining. The number of positive tumour cells was counted in 10 representative visual fields of each xenograft. Data are expressed as percent change (means±SD) compared to SCR group and represent four independent experiments (**P*<0.01 vs. SCR siRNA, Student *T*-test). Scale bar, 200 µm.

**Supplementary Figure 3**. Apoptosis was increased by *stathmin1* siRNA in xenografts. Xenograft tissues described in the legend of Supplementary Figure 1 was used for TUNEL assay. The number of positive tumour cells was counted in 10 representative visual fields of each xenograft. Data are expressed as percent change (means±SD) compared to SCR group and represent four independent experiments (**P*<0.01 vs. SCR siRNA, Student *T*-test). Scale bar, 200 µm.

**Table 1** Correlation between the expression of stathmin1 and clinical classification in gastric cancer

**P*<0.05 was defined as significant, Fisher’s exact test

**Table 2** Univariate and multivariate analysis of prognostic factors in the diffuse type of gastric cancer patients for recurrence-free survival

*CI=confidence interval; RR=relative risk
